# Impacts of COVID-19 Pandemic on Micro and Small Enterprises: Evidence From Rural Areas of Iran

**DOI:** 10.3389/fpubh.2022.844825

**Published:** 2022-05-26

**Authors:** Ahmad Yaghoubi Farani, Fatemeh Sepahvand, Saeed Gholamrezai, Hossein Azadi, Neda Nazemi

**Affiliations:** ^1^Faculty of Agriculture, Bu-Ali Sina University, Hamedan, Iran; ^2^Faculty of Agriculture, Bu-Ali Sina University, Hamedan, Iran; ^3^Faculty of Agriculture, Lorestan University, Khorram Abad, Iran; ^4^Department of Geography, Ghent University, Ghent, Belgium; ^5^Department of Engineering Systems and Environment, University of Virginia, Charlottesville, VA, United States

**Keywords:** rural business, adaptive behavior, passive behavior, COVID-19 crisis, Iran

## Abstract

Since 2020, the outbreak of the COVID-19 crisis has caused a great deal of social and economic damages to micro and small-scale enterprises (MSEs). This research examined the most common damages of this crisis in active and inactive rural MSEs and also assessed different kind of responses the managers and owners of theses MSEs have received dealing with these damages. The sample population of this study consisted of all managers of 72 active and 38 closed rural MSEs in the Dastjerd village, Hamedan, Iran. These MSEs were mainly garment small factories. This research utilized a mixed approach (quantitative-qualitative) to study the research objectives in depth. First, in qualitative part, semi-constructed interviews and field visits were done. Then, using quantitative, results of the qualitative section, previous studies and the existing literature, a researcher-made questionnaire was created. Based on qualitative part information through interviews, damages of rural MSEs during COVID-19 pandemic were categorized into three classes, including damages related to production, and financial and marketing issues. Also, two categories of managers' responses that could be labeled as passive and adaptive behavior were identified. Findings showed that active rural MSEs have taken more adaptive measures and tried to find appropriate ways to reduce or overcome damages. Active MSEs were mainly owned and managed collaboratively by more literate and experienced managers. Also results revealed that rural MSEs' managers reacted to different kinds of damages based on their ability, knowledge, and experience. Based on research results, managers' knowledge and skills can help them find more adaptive solutions to keep the firms stable and overcome damages. It can be concluded that COVID-19 pandemic has a great impact on rural MSEs and they need more financial support and managerial advice to overcome this kind of crisis situation.

## Introduction

In late 2019, the World Health Organization (WHO) reported the coronavirus outbreak in Wuhan, China, and later in March 2020 declared it as a global pandemic (1–4). This is the third widespread pandemic of the 21st century (5, 6) which led to a series of public health measures and interventions, including limitation on activities of education institutes, sport, cultural events, and non-essential retailers, to reduce the spread (7). Such interventions have adversely affected the global economy, leading to job losses and very high unemployment rates (8–12). Also declining direct international investment and consequently reduction of Gross Domestic Product (GDP) are consequences of these public health interventions in many countries around the globe (10, 13).

The COVID-19 crisis has far-reaching consequences for MSEs which include declining demand, increase costs, liquidity, and supply challenges. In general, some studies [e.g., (14, 15)] show that the adverse effect of the pandemic on MSEs has been more severe than larger units. Unemployment, financial losses, and layoffs are the most notable impacts of the economic shock of COVID-19. Generally, because of the scale of performance and limited financial resources, smaller firms are highly vulnerable and have low recovery capacity (15). Also, many smaller enterprises have not benefited enough from the government's relief plans due to their low awareness and limited access to information and resources (12, 16–18). These issues make them more vulnerable and less resilient in comparison to big corporations (19–21).

Recent studies (10, 15, 17) show that in the COVID-19 pandemic, MSEs have been dealing with declined demand, interrupted supply chain, export orders cancelation, shortage of raw materials, and disruption in transportation. And generally, because of low access to financial and managerial resources (12, 22), most of small enterprises are not ready to overcome the crisis damages.

Although there is no accurate information on the extent of the damages made to Iran's micro and small enterprises, the Iranian Unions Association estimated that the COVID-19 crisis has disrupted and damaged 57 business categories. They also attest the manufacturing enterprises, including garment production factories which have endured the most severe damages. Some MSEs in Iran fired at least one worker in the COVID-19 crisis and some managers do not expect their economic conditions to improve in the next 2 years (8). Also based on a study conducted by Institute of Trade Studies and Research in Iran, the COVID-19 crisis has affected the supply chain, demand and liquidity, labor supply, consumption of goods and services, and has reduced consumers' income (23). Although government support for MSEs is very important, in the end, the reactions and decisions made by MSEs' managers can have a significant impact on the future of their enterprises. Given the circumstances, MSEs may determine various responses such as decreased production due to lack of sales market, training of employees to go through the crisis period as well as fired workers (15, 24).

The COVID-19 crisis has also had a strong impact on rural communities. Rural communities, especially in developing countries, are the most vulnerable regions due to low income and high level of dependency on production resources (25). Based on this vulnerability, rural MSEs have more difficulties in crisis situations like the economic shock of COVID-19 (26). Due to the high hidden and real economic problems such as unemployment rate in rural communities (27), rural MSEs have great potential for job creation, unemployment reduction, and income increase through agricultural and non-agricultural activities (28). MSEs could also lead to the development of the local economy in rural areas (29, 30).

During the COVID-19 pandemic, many researchers tried to investigate the effects of COVID-19 pandemic on rural communities in their studies. Recent studies have mainly focused on the effects of the COVID-19 crisis on rural economic development, health, tourism, unemployment, mortality, community resilience, food security, etc. (31–37), but few studies have concentrated on the role of rural enterprises' managers and their competencies in making proper and quick decisions through this pandemic (38–40). Certainly, managers play a great role in making appropriate decisions in crises (41, 42). Also managers' competency is a key factor in crisis management (38). Therefore, it is important to know how business managers behave and make decision during these critical situations. In this research, we paid more attention to how rural MSEs' managers responded to different types of damages during theCOVID-19 pandemic, and how managers of active and closed rural SMEs behaved during this pandemic. Basically, the objectives of the current study include:

- Investigate different types of damages that rural MSEs have suffered more.
- Assessing managers' passive and adoptive behavior to crisis situation of the COVID-19 pandemic.
- Understand the personal and demographic characteristics of managers that affect their behavior.
- Comparing managers' responses of active and closed MSEs during pandemic.

## Methodology

### Study Area

**Figure 1:**
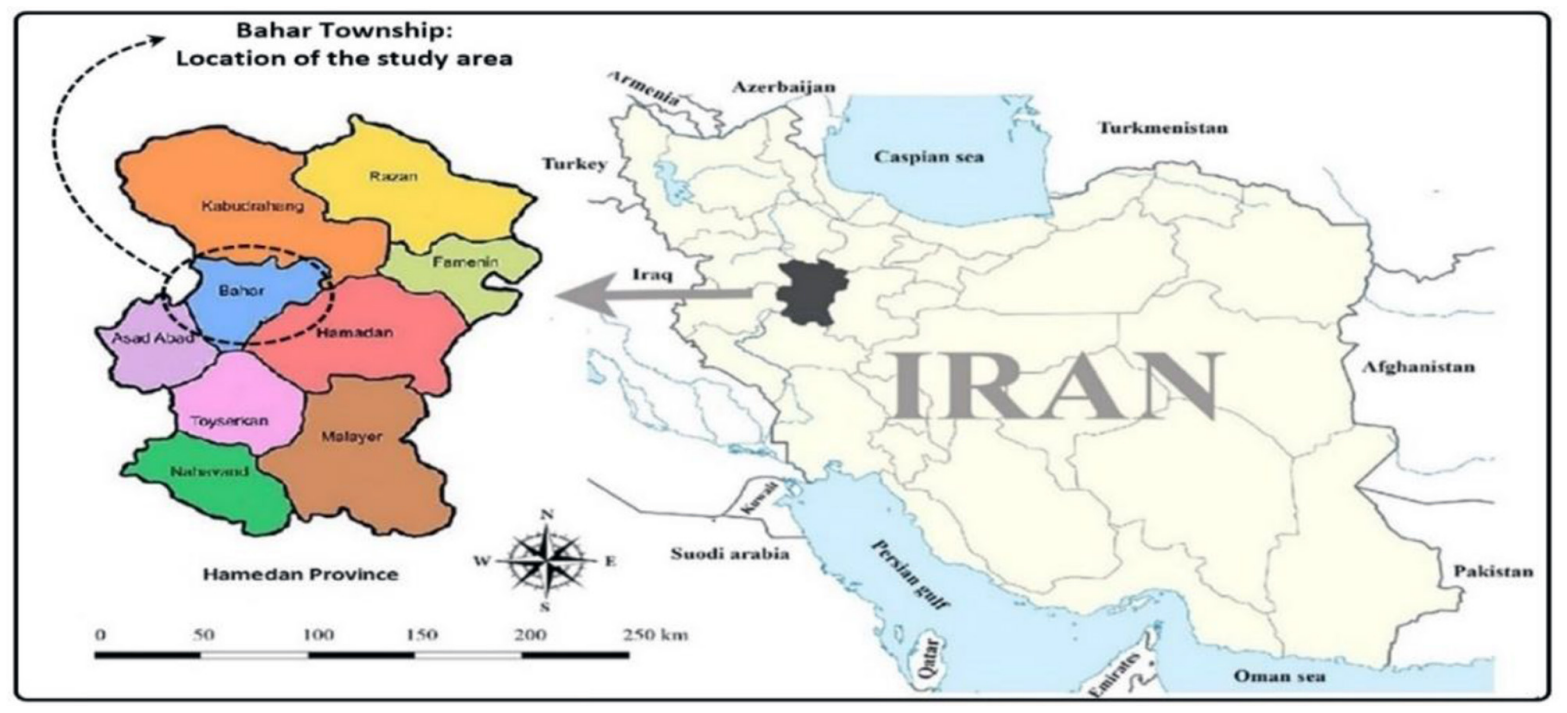
Geographical location of the study area.

The study area of the research is Dastjerd village, located in the Bahar county of Hamedan province. This village is an old and historical village with a population of more than 2,000 people. In Dastjerd village the main occupation of the people is agriculture, animal husbandry and tailoring. There are many semi-industrial sewing firms in this village that provided employment opportunities for many young rural people (Figure 1). Most of these sewing firms are specialized in Children's clothing production and more than 20% of national demand and 90% of Hamedan province demand for Children's clothing is being produced in this village.

### Data Collection Instrument and Analysis

#### In the Qualitative Part

This research utilizes a mixed approach (qualitative-quantitative) to study the research objectives in depth. In qualitative phase, a semi-constructed in-depth interview through field visit was conducted in the study area in January 2021. Twenty-four producers among the active and closed firms, and four related experts were selected through purposive sampling technique. This phase helped us to identify different types of damages rural MSEs have suffered more and also managers' behavior in response to the COVID-19 pandemic.

Interviews took place in the form of a guided interview. The interviewees were asked to talk about most damages their firms sustained and their responses to their confrontation with the crisis. These conversations were guided systematically to address the topic from different angles. Due to the prevalence of the COVID-19 crisis at the time of interviews, to keep social distancing, 11 face-to-face interviews and the rest of the phone calls were conducted to observe the social distance. The average time of phone call interviews was 30 min. All conversations were accurately recorded and analyzed using content analysis techniques. Content analysis is a systematic approach to compress a large number of texts and words into predefined content categories based on rules of coding (43). In this study, this approach was used to create damage categories based on coded keywords. With help of content analysis, we could categorize three classes of damages and also list the most common responses of the MSEs' managers in the COVID-19 crisis situation. In this part, a methodological triangulation technique was used for increasing the validity and reliability of findings. Triangulation involves the use of several kinds of methods or sources of information to obtain overlapping data and it is a tool for improving the accuracy, credibility, and validity of findings (44).

#### Analyzing Quantitative Data

The second part of the research was a quantitative study with a researcher-made questionnaire using the first part findings, previous studies, and the existing literature. Since no previous research study has been done on the impact of the COVID-19 crisis on rural businesses in Iran, the qualitative part was designed to understand the extent of damages and undertaken responses by affected MSEs. Ultimately, the questionnaire was developed through using these results and exploring the related literature. Therefore, the damages to the firms were identified through interviews in qualitative part and the related literature (14, 45). Then they were categorized into three classes including Damage Related to the Firm (DRF), Damages Related to Products (DRP), and Damages Related to Marketing (DRM).

The research population in this part consisted of all 110 managers of active and closed MSEs in the study area. The sample was taken by the census technique and all active (72 people) and closed (38 people) MSEs' managers were selected to gather data. Closed MSEs were those that were marked temporarily closed after the COVID-19 pandemic and had no activity at the time of field study (February 2021).

The research questionnaire consists of three sections:

1- DC: The demographic and background factors including age, gender, education, work experience, etc. (18 items).
2- Damage to MSEs, including DRP (four items), DRF (six items), and DRM (five items) (Table 1).
3- Response: This section examined managers' behaviors including two types of adaptive and passive responses (Table 2). The items were rated on a five-point Likert scale scoring system. The face and content validity of the questionnaire was confirmed by a panel of experts. The reliability of the questionnaire was also assessed with Cronbach's alpha (α) after a pilot test. In the Quantitative part, spss22 software was used to analyze data (Table 1).

**Table 1:** Description of different types of damages of MSEs in the COVID-19 pandemic.

**Category**	**α**	**Description of Damage**	**Reference(s)**
DRP		Decrease in firm's production in comparison to its routine operation	Self-developed
	0.92	A decline in the quality of products because of the shortage of raw materials	Self-developed
		Inability to trade as per unit's regular routine	Self-developed
		Reduced income and cash flow in comparison to pre-pandemic averages	Samantha (18) Ratten (14)
DRM		Inability to communicate with business customers	Samantha (18)
	0.93	The lack of access to market information in the short-term	Self-developed
		Loss of firm's customers	Samantha (18)
		The poor performance of the market in advertising the firm's products	Self-developed
DRF		Losing the savings and financial resources to cover unexpected costs	Samantha (18) Ratten (14)
	0.90	Inability to pay bank loans and mortgages	Faulkner et al. (46)
		Inability to pay the staffs' salaries	Self-developed
		Insufficient cash flow for covering the firms' daily costs	Vera (45)
		Stricter requirements for receiving loans	Self-developed

**Table 2:** Description of responses in adaptive and passive behaviors of MSEs' managers in the COVID-19 pandemic.

**Behavior category**	**α**	**Description of responses**
Passive		Adjustment of the labor force to lessen costs
behavior	0.82	Getting help from family members and relatives to reduce the labor cost
		Reducing the working hours of the firm to reduce costs
		Collaborating with other units to consolidate workshops and reduce costs
		Changing the career temporarily to provide the livelihood of family
		Attempting to migrate to town or cities to find other jobs
Adaptive behavior		Request a loan to cover the costs and compensate for financial losses
	0.73	Assigning or leasing part of the space and resources of the workshop to other businesses
		Producing alternative goods such as masks, and hospital guns with a better market in the crisis conditions
		Getting customized/ tailoring orders
		Consult with experts and informed people to find alternative and more suitable businesses
		Trying to learn new knowledge and skills to enter other businesses and markets

## Results and Discussions

### Demographic Characteristics

#### Active Firms

Demographic and personal characteristic analyses of 72 active firms in the village of Dastjerd determines that the age of the managers is between 21 and 57 years old, and the average is 40 years old. Active producers have 15 years of work experience on average. Generally, they have high school diplomas (40 people), 6 have a university degree, and 26 have only acquired basic literacy. In terms of work experience, the average is 15 years.

Ownership analysis of active firms shows that 48 firms have group (partnership) management, and 24 firms operate individually (sole proprietorship). Regarding learning new skills to improve business and management, 40 producers have not learned any new skill and knowledge dealing with complication of the COVID-19 crisis in the last 12 months, while 12 producers have acquired some new skills, and 18 producers have got some skills that are not directly applicable to the firm's management. Based on the data, at the time of the research, active firms have approximately 4 workforces, while at least two people have been laid off from 2020.

#### Closed Firms

Descriptive analysis of closed firms shows the age range of managers is between 19 and 55 years old, with an average of 35 years old. Most of them have high school diplomas (21 people), while 16 people have acquired basic literacy, and only 1 has a university degree. On average, they have 13 years of work experience in the firms. Regarding the type of ownership of the firms in this group, results reveal that 29 firms are sole proprietorships and managed individually, while only nine firms are partnerships (mostly family businesses) and managed collaboratively. Moreover, four workers have been laid off after closing firms. Table 3 briefly shows managers' personal and DC in two categories of active and closed firms.

**Table 3:** Mean and ranking of different damages in closed MSEs.

**Rank**		**Damages**	**Mean**	**S.D**
1	DRP	Decrease in firm's production in comparison to its routine operation	3.78	0.413
2		A decline in the quality of products because of the shortage of raw materials	3.65	0.480
3		Reduced income and cash flow in comparison to pre-pandemic averages	3.63	0.488
4		Inability to trade as per unit's regular routine	3.57	0.500
		**Mean total**	**3.66**	
1	DRM	The lack of access to market information in the short-term	3.73	0.446
2		Inability to communicate with business customers	3.63	0.541
3		Loss of firm's customers	3.57	0.500
4		Poor performance of the market in attracting the firm's products	3.52	0.556
		**Mean total**	**3.65**	
1	DRF	Loss of savings and financial resources to cover unexpected costs	3.28	0.611
2		Inability to pay back the bank loans and mortgages	3.18	0.691
3		Inability to pay the staffs' salaries	3.10	0.559
4		Stricter requirements for getting loans	2.76	0.589
		**Mean total**	**3.14**	

*DRM, Damage related to marketing; DRP, Damage related to production; DRF, Damage related to finance*.

### Damages Caused by COVID-19 Pandemic in Active and Closed MSEs

This section examines the type and extent of damages caused by the COVID-19 pandemic in active and closed firms and analyzes the most common adopted responses in active and closed MSEs separately.

#### Closed Firms

Table 3 presents the descriptive results of the analysis on closed firms. The results indicate that the most extensive damages to closed firms are related to DRP and DRM, with average scores of 3.66 and 3.65, respectively. Also, DRF ranked third in magnitude, with an average score of 3.14 (out of 5 points).

Ranking of the damages (third column) reveals that the decline in production was the first and foremost damage to the closed firms. It means that these firms could not keep the production volume which has led them to fall behind in the market cycle. The second disadvantage of closing companies is related to sales and marketing that can be associated with the managers' lack of access to information and inability to predict the short-term impact of the COVID-19 crisis on the market. Therefore, lack of foresight knowledge and ineffective communication customers of companies could lead to loss of customers and eventually complete closure of the unit. Generally, damages to production and marketing are highly correlated to the lack of marketing opportunities that would directly affect the unit's production capacity.

The descriptive results of the last part also confirmed that the managers of the closed firms were mainly less educated compared to active firms. It might have affected their capability of observing the market changes and impacted their analytical thinking and strategic decision-making skills. Finally, the results of financial damages also determine that the closed firms have had fewer financial revenues due to damages related to production and marketing which led to “Insufficient cash flow for covering the firms' daily costs.” As a result, these firms could not pay salaries and had to lay their workers off. Results (Table 4) confirm that the number of laid-off workers in these firms is two times more than workers fired in the active firms.

**Table 4:** Personal and demographic characteristics of managers in active and closed MSEs.

**Descriptive components**	**Active**	**Closed**
Number of firms	72	38
Average age (year)	40	35
Average workforce	4	0
Ave. no. of Workers fired	2	4
Work experience (year)	15	13
Level of education	Limited ability to read and write: 26 (%36/1) High school: 40 (%55/6) College degree: 6 (%8/3)	Limited writing and reading: 16(%42/1) High school: 21(%55/3) College degree: 1(%2/6)
Type of ownership	Partnership: 48 (%66/7) Sole proprietorships: 24(%33/3)	Partnership: 9(%24/7) Sole proprietorships: 29(%76/3)

In the following, the responses are examined in closed firms (Table 5). The responses are classified as adaptive and passive. Research results show that the average of passive and adaptive responses is equal (4.30), which means the closed firms have taken as many passive measures as adaptive ones. At the beginning of the pandemic, as the orders started to be canceled and new orders declined, the initial response of these firms was “temporarily change of job to provide for the family livelihood.” At that time, the temporary shutdown was mandatory, and by March 2020, all unnecessary businesses like garment factories had to stop their activities.

**Table 5:** Mean and ranking of adaptive and passive behaviors in closed MSEs.

**Rank**		**Behaviors**	**Mean**	**S.D**
1	Adaptive behavior	Producingalternative goods such as masks, hospital guns with a better market in the crisis conditions	3.47	0.903
2		Getting customized/ tailoring orders	3.33	1.363
3		Request a loan to cover the costs and compensate for financial losses	1.47	1.64
4		Consult with experts and informed people to find alternative and more suitable businesses	0.84	1.370
5		Trying to learn new knowledge and skills to enter other businesses and markets	0.75	1.31
6		Assigning or leasing part of the space and resources of the workshop to other businesses	0.37	1.05
		**Mean total**	**1.87**	
1	Passive behavior	Reducing the working hours of the firm to reduce costs	1.90	1.365
2		Receiving help from family members and relatives to reduce the labor cost	1.47	1.678
3		Adjustment of the labor force to lessen costs	0.91	1.65
4		Changing the occupation temporarily to provide the livelihood of family	0.76	1.65
5		Attempting to migrate to town or cities to find other jobs	0.23	0.759
		**Mean total**	**1.05**	

When the temporary shutdown ended up and the firms restarted their activities, some of these firms chose to “reduce their labor force to decrease the costs,” since they had been closed for a while and did not have enough revenue. Alternatively, some other firms chose to “Reduce the firm's working hours to overcome the costs.” They made these responses while they had no vision of what would happen in the following months. These 38 closed units were solely relying on orders coming from the capital, Tehran. Consequently, without receiving new orders during the pandemic, they could not continue their activities and had to shut down.

The results also showed that these firms had to take passive measures temporarily, and the adaptive responses were not successful. Therefore, most of these firms' managers and workers had to go to nearby cities to find civil service jobs such as doorman, salesman, security, cashier and similar job positions. Also, the responses analysis showed that closed firms also tried to make adaptive measures, but none of these decisions could save these firms from closure. It might be because of the coincidence of the pandemic with a dramatic increase in raw materials prices. For instance, the yarn price doubled and, in some cases tripled up. In this condition, firms even were not able to fulfill the previous orders.

Based on the research results, the main features of closed firms' managers include the lack of skills and knowledge other than just sewing, lack of alternative financial resources (i.e., second job, saving accounts) to manage workshop costs, and lack of interest in requesting loans. It should be noted that most of these firms were sole proprietorships and individually managed and had no business advisor.

#### Active Firms

The results of damage analysis on active firms (Table 6) indicate that they have also sustained many damages over the past year. The most incurred damages relate to sales and marketing with an average score of 2.8 followed by production-related and financial-related damages with average scores of 2.63 and 2.21, respectively. The most critical identified damage to active firms was the damage related to sales and marketing. Active firms have been dealing with “the lack of access to market information in the short-term.” Since this crisis is an unprecedented event, both active and closed firms have not been able to predict the market even for a few months ahead. This uncertainty has also caused “inability to communicate with business customers” and eventually led to “loss of firm's customers.” Damage to production rate is the second-ranked damage in terms of frequency and intensity. Lack of market certainty and security and the ongoing loss of customers have led to “reduced income and cash flow” and “decreased firm's production.” Ultimately, active firms have suffered financial damages; however, it is less extensive than the other two categories. Going through marketing and production damages significantly affected their financial conditions and has led to “losing the savings and financial resources to cover unexpected costs.” Due to loss of financial resources, covering the daily costs of units and loan payments have been challenging for these firms.

**Table 6:** Mean and ranking of different damages in active MSEs.

**Rank**		**Damages**	**Mean**	**S.D**
1	DRM	Lack of access to market information in the short-term	2.86	0.860
2		Loss of firm's customers	2.69	0.798
3		Poor performance of the market in attracting the firm's products	2.56	0.885
4		Inability to communicate with business customers	2.51	0.919
		**Mean total**	**2/80**	
1	DRP	Reduced income and cash flow in comparison to pre-pandemic averages	2.69	0.987
2		Decrease in firm's production in comparison to its routine operation	2.68	0.801
3		A decline in the quality of products because of the shortage of raw materials	2.58	0.726
4		Inability to trade as per unit's regular routine	2.58	0.851
		**Mean total**	**2.63**	
1	DRF	Losing the savings and financial resources to cover unexpected costs	2.29	0.680
2		Insufficient cash flow for covering the firms' daily costs	2.41	0.745
3		Stricter requirements for getting loans	2.16	0.650
4		Inability to pay back the bank loans and mortgages	2.09	0.479
5		Inability to pay the staffs' salaries	2.09	0.653
		**Mean total**	**2.21**	

*DRM, Damage related to marketing; DRP, Damage related to production; DRF, Damage related to finance*.

After analyzing the extent of damages, the responses taken over last year (2020) by active firms were examined (Table 7). In the early stages of the epidemic, when government restrictions were increasing, executives of active firms changed their product lines to “produce better-market alternatives in crisis situations such as masks, hospital guns, and custom tailoring” that could support their help their livelihoods during a crisis. Having said that, it is evident that these firms were actively looking for alternative markets to avoid shutting down their production lines. The active firms have taken the least passive responses. Most of these firms are partnerships and are being managed collaboratively. This fact might have impacted the type of responses that they undertook during the COVID-19 crisis. For example, some firms initially decided to “collaborate with other units to consolidate workshops and reduce costs. This shows that these firms have been more open to collaborative decision-making. Then, they chose “reducing the firm's working hours to reduce costs” and “getting help from family members and relatives to reduce the labor cost” as subsequent passive responses to fight the consequences of the crisis.

**Table 7:** Mean and ranking of adaptive and passive behavior in active MSEs.

**Rank**		**Behaviors**	**Mean**	**S.D**
1	Passive behavior	Changing the occupation temporarily to provide the livelihood of family	4.34	1.02
2		Adjustment of the labor force to lessen costs	3.89	1.18
3		Reducing the working hours of the firm to reduce costs	3.68	0.873
4		Getting help from family members and relatives to reduce the labor cost	2.55	1.94
5		Attempting to migrate to town or cities to find other jobs	2.44	2.16
		**Mean total**	**3.38**	
1	Adaptive behavior	Trying to learn new knowledge and skills to enter other businesses and markets	3.81	1.64
2		Producing alternative goods with a better market in the crisis conditions such as masks, hospital guns	3.47	2.02
3		Consult with experts and informed people to find alternative and more suitable businesses	3.15	1.77
4		Assigning or leasing part of the space and resources of the workshop to other businesses	3.05	2.06
5		Request a loan to cover the costs and compensate for financial losses	2.55	1.92
6		Getting customized/ tailoring orders	2.13	1.96
		**Mean total**	**3.04**	

#### Differences Between Active and Closed MSEs

Studying the differences between active and closed firms reveals that active firms have generally encountered less financial, production, and marketing damages since the averages of all three types of damages are <3 (out of 5). It confirms more resilience of these firms compared to closed firms. Additionally, other results (the second column of Table 8) verify that active firms have taken fewer responses (either adaptive or passive). Moreover, there is a statistically significant difference between these two types of producers regarding incurred damages and undertaken responses.

**Table 8:** Comparison of active and closed firms in sustained damages and token responses.

	**Type**	**Mean**	**Mean rank**	**Mann-Whitney *U***	**Sig. (2-tailed)**
DRP	Active	2.6	38.91	357.000	0.000
	Closed	3.66	75.35		
DRF	Active	2.21	37.34	253.500	0.000
	Closed	3.14	78.15		
DRM	Active	2.80	40.20	442.500	0.000
	Closed	3.65	73.04		
PB	Active	3.38	87.16	211.000	0.000
	Closed	1.05	38.79		
AB	Active	3.04	77.26	498.500	0.000
	Closed	1.87	44.01		

*AB, Adaptive behaviors; PB, Passive behaviors; DRF, Damage related to finance; DRP, Damage related to production; DRM, Damage related to marketing*.

## Conclusion

This study investigated the major damages that rural MSEs have faced due to the limitation and complication of the COVID-19 pandemic in Dastjerd village in Hamedan province, Iran. Also, it assessed different kinds of responses the managers and owners of these MSEs have received while dealing with these damages. All rural MSEs surveyed in this research were in the field of sewing garments. During the COVID-19 crisis, some of them (34.5%) had to stop their activity and close their workshops. Accordingly, in order to compare the most common damages and managers' behavior in two groups of active and closed MSEs, both groups have been considered with a mixed paradigm of qualitative and quantitative research. Research findings in the qualitative part contributed to our knowledge about different types of damages inflicted upon both groups of active and closed MSEs due to the COVID-19 limitation. Based on the qualitative part and information through interviews, we categorized all kinds of damages into three classes including DRF, DRP, and DRM. It revealed that, during the COVID-19 pandemic and its' challenges, rural MSEs were vulnerable due to the poor financial strengths and managerial skills and limited sales market and communication networks. Mueller et al. (25) and Malherbe et al. (26) also found that rural small businesses especially in developing countries were more vulnerable during COVID-19 pandemic. Workers' illness, workshop closure, lack of demand, lack of liquidity, and also rising costs were the most common causes that led the MSEs facing other damages. Shaf et al. (10); Bansal (15), and Eggers (17) also found that MSEs have been dealing with declined demand and interrupted supply chain through COVID-19. The results showed in both closed and active MSEs, the most damages were related to sales and marketing issues. However, the level of all damages was lower in active firms.

In qualitative phase, different kinds of managers' decisions in the COVID-19 crisis situation were also identified. According to information obtained in this regard, two categories of responses were identified, that could be labeled as passive behavior and adaptive behavior. Temporarily changing the occupation, labor adjustment, and reducing the working hours to reduce costs are the samples of passive behavior. Also in adaptive behavior, trying to learn new knowledge and skills to enter other businesses and markets, request loans to cover the costs, and producing alternative goods with a better market were mentioned. Based on the findings, active MSEs have received more adaptive responses and tried to find appropriate solutions to reduce or overcome damages. Furthermore, results revealed that MSEs' managers reacted differently based on their abilities, knowledge and experiences. Some MSEs decided to shut down their firms after a short period of time due to poor financial capacity and lack of skills and information in crisis management. Conversely, managers with more knowledge and experience were better able to manage this crisis and prevent the closure of their firms. This can indicate the importance of managers' knowledge and skills during the crisis. Dirani et al. (38) and Al-Dabbagh (39) had also emphasized on managers competencies during crisis like COVID-19 pandemic.

Research findings also highlighted that there is a difference between ownership form among two groups of active and closed MSEs so that the ownership of most active MSEs (66.7%) were shared ownership, while only 24.7% in closed MSEs were shared ownership and most of them (76.3%) were sole proprietorships. Accordingly, it can be concluded that shared firms may have more capacities, especially for financial support to overcome difficulties in crisis situations.

Finally, as stated in our article, the COVID-19 pandemic has had a great impact on rural MSEs and some of MSEs' managers had to close their firms because of different damages related to the process of production, marketing, and financial issues. It can mainly be concluded that rural MSEs need more financial support and managerial advice to overcome this kind of crisis situation. Based on research results, managers' knowledge and skills can help them find more adaptive solutions to keep their firms stable and overcome damages. Therefore, in addition to government support, training and development of managers' capabilities and crisis management skills are essential. This study helps government agencies identify the common damages to rural MSEs and the success rate of their adaptive behaviors in dealing with this pandemic.

## Data Availability Statement

The raw data supporting the conclusions of this article will be made available by the authors, without undue reservation.

## Author Contributions

AY and SG designed and directed the original research. FS collected data and managed the interviews. AY and FS helped in analyzing data and developed the main text. NN helped in translating some part of text from Persian to English. HA helped in scientific editing. AY and HA provided instruction and comments for enriching the main text. All authors contributed significantly to the creation of this manuscript and have read and approved the final manuscript.

## Conflict of Interest

The authors declare that the research was conducted in the absence of any commercial or financial relationships that could be construed as a potential conflict of interest.

## Publisher's Note

All claims expressed in this article are solely those of the authors and do not necessarily represent those of their affiliated organizations, or those of the publisher, the editors and the reviewers. Any product that may be evaluated in this article, or claim that may be made by its manufacturer, is not guaranteed or endorsed by the publisher.
