# Supplementary material for: Impacts of COVID-19 Pandemic on Micro and Small Enterprises: Evidence From Rural Areas of Iran
Source: Front Public Health. 2022 May 26;10:844825. doi: 10.3389/fpubh.2022.844825 (PMC9204275; doi:10.3389/fpubh.2022.844825)
Supplement: Supplementary file 1 [file Table_1.DOCX]

**Graphical abstract**

**Covid-19 Pandemic**

**Effects on rural MSEs**

Damages related to products (**DRP**)

Damages related to marketing (**DRM**)

Damage related to finance (**DRF**)

Passive & active responses of MSEs’ managers

Demographic characteristics of active and closed MSEs

**Results:**

**Mixed approach methodology**

**Qualitative & Quantitative**

**Research objectives:**

- **Investigate different damages on MSEs**
- **Assessing passive & adoptive behaviors**
- **Comparing responses of active & closed MSEs**
